# One-Year Quality of Life Outcomes of Delayed Unilateral Autologous Breast Reconstruction and Associated Patient Factors

**DOI:** 10.1016/j.jpra.2024.10.014

**Published:** 2024-11-01

**Authors:** Michael S. Mayr-Riedler, Sebastian Holm, Iliana Aristokleous, Bart de Vries, Andres Rodriguez-Lorenzo, Tua Riilas, Maria Mani

**Affiliations:** a Department of Surgical Sciences, Faculty of Medicine, Uppsala University, Uppsala, Sweden; b Department of Head, Neck and Reconstructive Plastic Surgery, Örebro University Hospital, Örebro, Sweden; c School of Medical Sciences, Örebro University, Örebro, Sweden; d Department of Surgery, Uppsala University Hospital, Uppsala, Sweden; e Department of Plastic and Reconstructive Surgery, Radboud University Medical Center Nijmegen (Radboudumc), GA Nijmegen, The Netherlands; f Department of Plastic and Maxillofacial Surgery, Uppsala University Hospital, Uppsala, Sweden

**Keywords:** Breast reconstruction, Autologous, DIEP, PROM, Breast-Q

## Abstract

**Introduction:**

As breast cancer survival rates improve, the long-term quality of life (QoL) has become increasingly important. With a significant number of patients still undergoing mastectomy and experiencing its well-known negative impacts on QoL, breast reconstruction aims to mitigate this by restoring body integrity. This study aimed to evaluate the changes in QoL and satisfaction in patients after breast reconstruction and influence of various patient-related factors.

**Methods:**

Patients who underwent delayed unilateral deep inferior epigastric perforator (DIEP) breast reconstruction at a single institution between January 2016 and April 2019 were surveyed. QoL was assessed using the BREAST-Q questionnaire preoperatively and one year postoperatively. Scores were compared between the time points, and regression analysis was conducted to identify the influence of age, body mass index, economic status, education level, and relationship status on QoL.

**Results:**

Among the 93 patients, 55 completed the preoperative and one-year postoperative BREAST-Q questionnaires (response rate: 59%). Postoperative QoL scores significantly increased for the domains “satisfaction with breasts,” physical well-being chest,” “sexual well-being,” and “psychosocial well-being” (p<0.001). The scores for the domain “physical well-being abdomen” remained unchanged one year postoperatively. Higher education correlated with greater satisfaction with the breasts. Lack of an intimate partnership was identified as a significant negative factor for poorer psychosocial well-being.

**Conclusions:**

Delayed unilateral DIEP breast reconstruction significantly enhances QoL and breast satisfaction one year postoperatively without causing long-term physical discomfort at the donor site. Education level and relationship status significantly affect the postoperative outcomes. Tailored preoperative counseling and psychosocial support are essential to maximize patient satisfaction and well-being following reconstruction.

## Introduction

Given that breast cancer mortality rates have decreased in the recent decades, quality of life (QoL) of breast cancer survivors has become increasingly significant.^1^ Breast cancer negatively impacts a patient's QoL, as shown by studies indicating decreased QoL scores following initial treatment and during long-term survivorship.^2^, ^3^, ^4^ Patients with breast cancer may suffer from feelings of fear and concerns about cancer, negative body image, self-evaluation, and reduced emotional, social, and sexual functioning.^5^ The negative effects were especially strong in patients who underwent mastectomy.^6^^,^^7^

Over the past few decades, there has been a shift toward less invasive surgical treatments for breast cancer, with breast-conserving surgery now being the standard of care for early-stage breast cancer.^8^ However, a significant number of patients continue to undergo mastectomy. Mastectomy rates fluctuate considerably over time and across different regions, with recent studies from larger multicenter cohorts reporting rates ranging from 13% to 40%.^9^, ^10^, ^11^, ^12^ The literature suggests that patients undergoing breast reconstruction exhibit significantly higher QoL scores compared to those undergoing mastectomy without reconstruction or even those after breast-conserving therapy.^13^, ^14^, ^15^

Several studies, comparing patient-reported outcomes of various breast reconstruction techniques, have shown that autologous reconstructions offer significantly higher patient satisfaction and superior long-term outcomes than implant-based reconstructions.^16^, ^17^, ^18^, ^19^, ^20^ Additionally, abdominal-based flaps have been shown to outperform other routinely used autologous flaps and are regarded as the gold standard for autologous reconstructions.^21^^,^^22^

Although patients undergoing mastectomy and immediate breast reconstruction grapple with the general psychosocial burden of cancer, those undergoing delayed reconstruction also struggle with the psychosocial distress and physical discomfort of living with a flat chest. Preoperative baseline data from patients opting for delayed autologous reconstruction provide better understanding of the direct effects of breast reconstruction on individual QoL. However, the literature lacks such data. Previous studies that incorporated preoperative QoL values either did not exclusively involve patients undergoing delayed reconstructions, had inadequate or short-term follow-up, or did not employ breast reconstruction-specific validated questionnaires.^23^, ^24^, ^25^, ^26^

Hence, the objective of this study was to examine the pre- and postoperative QoL and satisfaction levels in patients who underwent unilateral delayed deep inferior epigastric perforator (DIEP) breast reconstruction after therapeutic mastectomy. Additionally, we aimed to assess the influence of patient-related factors on patient-reported outcomes.

## Methods

A retrospective review of partially prospectively collected data of patients who underwent delayed unilateral DIEP breast reconstruction between January 2016 and April 2019 was performed at a university hospital in Sweden. The following exclusion criteria were defined: age under 18 years, risk-reducing mastectomy, prior reconstructive procedures, and recurrent or new breast cancer. Informed consent was obtained from all patients. The study was approved by the institutional research ethics committee (Dnr: 2014/354/1). The study and manuscript preparation were conducted in accordance with the STROBE guidelines for cohort studies.

### Clinical and demographic data

Patient clinical characteristics were extracted from the electronic patient files. These included age, body mass index, smoking history, comorbidities, date of cancer diagnosis, date of reconstructive surgery, and complications. Complications included vascular flap compromise, flap necrosis (partial or full), wound complications, and hematoma. Complications requiring surgical revision under general anesthesia were considered major complications.

Demographic data including economic status, education level, and marital status were obtained from a questionnaire regarding sociodemographic characteristics, which was routinely filled out preoperatively by all patients together with the preoperative BREAST-Q questionnaire. The postoperative BREAST-Q questionnaire was sent out to all patients one year postoperatively after the breast reconstruction.

### Breast-Q

The Swedish versions of the pre- and postoperative BREAST-Q questionnaire (Reconstruction module; Version 1.0) were used. The BREAST-Q questionnaire (Reconstruction module) is a validated tool for measuring QoL and satisfaction among breast reconstruction patients.^27^ The domains analyzed in this study are “satisfaction with breasts,” “physical well-being – chest,” “physical well-being – abdomen,” “sexual well-being,” and “psychosocial well-being.” Responses from the BREAST-Q questionnaires were transformed to a Q-score using Q-score software (Memorial Sloan Kettering Cancer Institute, New York, Version 1.6). The Q-score ranged from 0 to 100, where higher values indicate higher levels of QoL and satisfaction.

### Statistical analysis

Descriptive statistics were used to present patient, clinical, and sociodemographic characteristics and the Q-scores. Continuous values were expressed as means and medians with interquartile ranges, and discrete values were expressed as counts (%). Parametric analyses were used owing to the normally distributed nature of data based on a test for normality. Therefore, paired t-tests were used to test the statistical difference between the pre- and one-year postoperative Q-scores for each domain.

Based on the included number of patients, 5 patient-related factors were analyzed in relation to the one-year postoperative Q-scores for each domain: age, BMI, economic status (A=low (<100,000 Swedish Kronas (SEK)/year); B=low to medium (100,000–250,000 SEK/year); C=medium to high (250,000–400,000 SEK/year); D=high (>400,000 SEK/year)), education level (primary school, high school, and university), and marital status (married, relationship, single, and widow). To determine the influence of these 5 patient-related factors on the postoperative Q scores, univariable and multivariable linear regression analyses were performed. Furthermore, the patient-related factors were tested through stepwise regression analysis. A p-value <0.05 was considered statistically significant and confidence intervals were calculated to the 95% level. Analyses were performed using R (version 4.2.2, The R Project for Statistical Computing).

## Results

### BREAST-Q

Out of the 93 patients who underwent unilateral delayed DIEP-reconstruction, 55 patients responded to preoperative and one-year postoperative BREAST-Q questionnaires (response rate of 59%). The mean age of the responders at the time of breast reconstruction was 53.5 years (range: 35-70 years). The mean time between breast cancer diagnosis and reconstruction procedure was 32 months (4-162 months). Further patient demographics are outlined in Table 1.

**Table 1:** Patient Demographics (n=55).

Characteristics	Number of patients (%)
**BMI, kg/m^2^**	
18.5–24.9	16 (29)
25–29.9	31 (56)
30–34.9	4 (7)
Missing	4 (7)
**Smoking history**	
Active Smoker	3 (5)
Past Smoker	18 (33)
Never smoked	29 (53)
Missing	5 (9)
**Other long-term comorbidities**	
Yes	23 (42)
No	32 (58)
**Highest education**	
Primary School	4 (7)
High School	22 (40)
University	29 (53)
**Annual income (SEK)**	
A=low (<100,000 SEK/year)	2 (4)
B=low to medium (100,000–250,000 SEK/year)	11 (20)
C=medium to high (250,000–400,000) SEK/year	25 (45)
D=high (>400,000 SEK/year)	17 (31)
**Postoperative complications**	
Major (requiring surgical revision)	
Venous flap compromise	3 (5)
Partial flap necrosis	1 (2)
Minor (nonsurgical management)	1 (2)
Wound complication	12 (22)
Hematoma	1 (2)
**Additional surgery** (i.e., nipple reconstruction)	
Yes	50 (91)
No	5 (9)

Abbreviations: SEK, Swedish Krona; BMI, body mass index.

**Figure 1:**
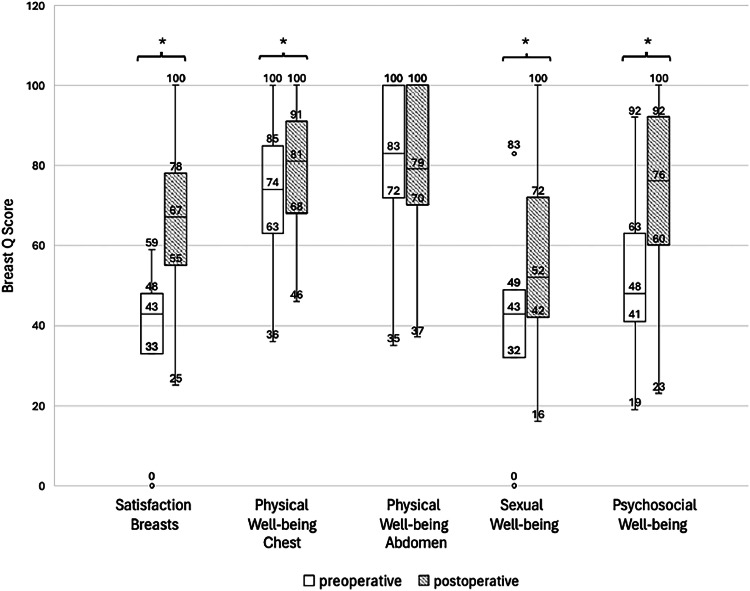
Box plots indicating preoperative (white boxes) and postoperative (gray boxes) Breast-Q scores among the five subdomains (*=p<0.001 based on paired t-tests).

The one-year postoperative Q-scores were significantly higher (p <0.001) than the preoperative Q scores across all domains except for "physical well-being – abdomen," which showed no change (80 preoperative and 82 postoperative, p=0.835). (Figure 1)

### Patient-related factors analysis

#### Satisfaction with breasts

**Figure 2:**
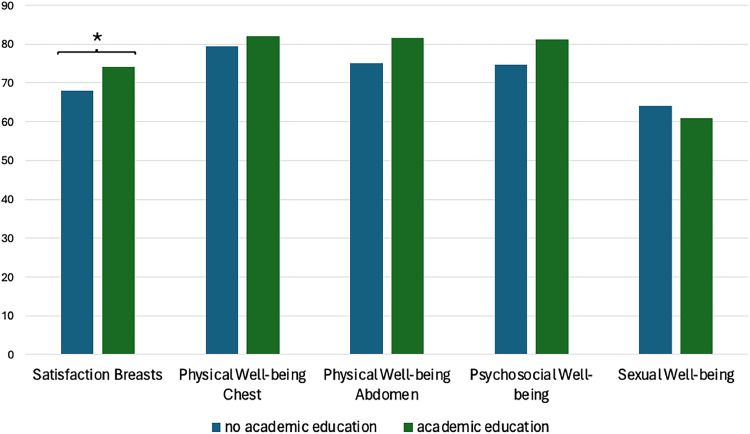
Bar chart illustrating the mean postoperative Breast-Q scores for patients with no academic education (primary school or high school) (blue bars) and patients with academic education (university degree) (green bars) (*=p<0.05 based on multivariable regression analysis).

Academic education was significantly associated with higher Q-score on the domain “satisfaction with breast” in the univariate regression analysis (b=10.13, p=0.018). Academic education was also an individual factor for higher satisfaction with breast in multivariable analysis (β=12.83, p=0.017). (Figure 2)

#### Physical well-being of the chest

**Figure 3:**
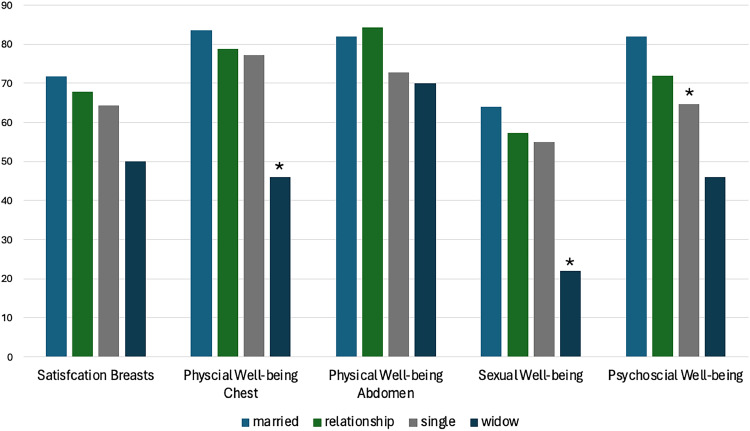
Bar chart illustrating the mean postoperative Breast-Q scores according to the patients’ relationship status (light blue bar: married, green bar: relationship; gray bar: single; dark blue bar: widow). Asterisk (*) indicating significant (p<0.05) factors based on the multivariable or stepwise regression analysis.

Univariate regression analysis showed that medium to high economic status (category C, income of 250,000–400,000 SEK/year) was associated with significant higher Q-score in the domain “physical well-being of the chest” (b=10.32, p=0.043), while the relationship status “widow” was associated with significantly lower Q-score (b=−37.67, p=0.012). Relationship status “widow” was an individual factor for lower physical well-being of the chest in multivariable regression analysis (β=−29.54, p=0.015). (Figure 3)

#### Physical well-being of the abdomen

None of the assessed parameters was significantly associated with the Q-scores in the domain “physical well-being of the abdomen.”

#### Sexual well-being

Patients who were widows trended toward lower Q-scores on sexual well-being in the univariate analysis (b=−41.50, p=0.05) and the status “widow” was a significant individual factor for lower sexual well-being in the stepwise regression analysis (ß=−27,40, p=0.018). Higher age was also a significant individual factor for lower sexual well-being in the multivariable regression analysis (β=−0.96, p=0.049).

#### Psychosocial well-being

Patients with academic education trended toward higher Q-scores on the domain psychosocial well-being (b=10.52, p=0.05), whereas singles had significantly lower psychosocial well-being (b=−17.46, p=0.01) in the univariate analysis. The relationship status “single” also was found to be a significant individual factor for lower psychosocial well-being in the stepwise regression analysis (ß=−17.40, p=0.01).

The factor “BMI” showed no significant association with any domain in the one-year postoperative Q-scores.

## Discussion

The key outcome measure following breast reconstruction is the patient-reported satisfaction with the breast and QoL. Although studies comparing different reconstructive methods provide valuable evidence for decision-making regarding the surgical techniques, understanding the impact of breast reconstruction on restoring well-being and QoL through enhanced body integrity necessitates the knowledge of preoperative baseline values. The current study shows that delayed unilateral DIEP breast reconstruction significantly enhances preoperative QoL by increasing the satisfaction with breasts and overall well-being one-year postsurgery.

The significance of preoperative values in assessing the effect of unilateral delayed breast reconstruction becomes particularly evident when comparing our findings to normative BREAST-Q data. Compared to the recently published normative reconstructive BREAST-Q scores of 400 Swedish women, the preoperative baseline values in our study population were considerably lower among all domains except for “physical well-being abdomen.”^28^ These results were expected to a certain degree and underscore the negative effects of breast cancer and mastectomy on the patients’ QoL. Remarkably, the scores one year after reconstruction were higher than the normative reconstructive BREAST-Q scores in all domains except for the domain “physical well-being chest” for which the scores also significantly increased after reconstruction but did not reach the level of normative data.

Notably, the domain “physical well-being abdomen” remained unchanged one year postoperatively. The abdominal area is not affected by the initial disease or breast cancer therapy itself but only serves as a donor site during reconstruction. This explains that the preoperative scores in the domain “physical well-being abdomen” were generally high and equaled that of published normative data.^28^ Achieving postoperative values for physical well-being at the donor site that conform to preoperative values and normative data can be interpreted as a positive outcome and underscores the low morbidity of abdominal-based perforator flaps. Our findings align with those of previous outcome studies on donor site morbidity following abdominal-based perforator flaps, notably those with extended follow-up durations of 3 years or more.^29^^,^^30^ However, Zhong et al. reported significantly reduced physical well-being scores at the 3-month mark postsurgery.^23^ Therefore, considering our results alongside the existing literature, patients can be advised that physical discomfort during the surgical site healing process may persist for several months but is anticipated to diminish within the first postoperative year.

Multiple patient-related factors were identified to impact the outcome after delayed unilateral DIEP breast reconstruction. In our study, relationship status and education level were found to have the strongest impact, influencing multiple domains of the BREAST-Q. The literature provides evidence that marital status influences breast cancer survival through association with cancer stage at diagnosis and compliance with adjuvant therapy.^31^, ^32^, ^33^, ^34^, ^35^ Sergesketter et al. further showed that reconstruction after mastectomy was more likely to occur in patients in marriages or partnerships.^36^ Our study demonstrated that patients without a spouse or intimate partnership have worse psychosocial and sexual well-being as well as physical well-being of the chest after reconstruction. These findings show that the positive effects of relationship support go beyond acute breast cancer treatment.

Postoperative satisfaction after breast reconstruction is strongly related to preoperative expectations; however, the initial expectations of patients undergoing breast reconstruction may be unrealistic.^37^ Previous studies indicate that providing detailed preoperative information reduces postoperative decision regret of undergoing reconstruction and increases postoperative satisfaction.^38^, ^39^, ^40^ We found a significant positive association of higher education and satisfaction with breasts. The rationale behind this association might be that patients with academic education do better in acquiring information preoperatively and therefore have better understanding of the treatment plan and more realistic expectations of the postoperative results.

At our institution, patients benefit from continuous support provided by a designated contact nurse, supplemented by information offered through patient groups and individual preoperative counseling. However, our results demonstrate that despite these efforts, patient-related factors that cannot be directly modified significantly influence the final patient-reported outcome.

There is an ongoing discussion regarding the optimal timing (immediate or delayed) for breast reconstruction. To ensure a homogeneous study group in terms of timing, our study exclusively evaluated outcomes following delayed breast reconstruction. Previous studies have highlighted the differences in overall complication rates based on the timing of breast reconstruction, though no significant difference in overall PROMs related to timing have been identified.^26^^,^^41^, ^42^, ^43^, ^44^ However, it remains possible that variations in the subdomains of PROMs may exist depending on the surgical timing, influenced by the different preoperative expectations and postoperative satisfaction among patients who undergo immediate reconstruction compared to those who live without breasts for a certain period before undergoing delayed reconstruction.

The absence of a control group consisting of patients with breast cancer who underwent mastectomy without breast reconstruction represents a potential limitation of our study. It can be argued that a baseline improvement occurs in the QoL after acute breast cancer treatment over time, regardless of whether breast reconstruction is performed. In our study, the average period between cancer diagnosis and reconstruction was 32 months. Therefore, any potential baseline changes in QoL should be evident in the preoperative values, thus limiting bias. The follow-up in our study is relatively short-term (one year) and the results may change over time. A further limitation of our study is the modest response rate, which may introduce potential selection bias.

## Conclusion

Our findings indicate that delayed unilateral DIEP breast reconstruction effectively mitigates the adverse effects of breast cancer and mastectomy on the overall well-being and satisfaction with breasts, without causing persistent physical discomfort at the donor site. This highlights the importance of breast reconstruction as a fundamental component of a comprehensive approach to breast cancer treatment and raises concerns about the "going flat" trend from a medical perspective.^45^ The findings of this study support the importance of personalized information and the patients’ educational levels and of offering psychosocial support, particularly among patient groups with limited support from partners and families.

## Funding

None.

## Conflict of Interest

None.

## Ethical approval

The study was approved by the institutional ethical committee (Dnr: 2014/354/1).
